# Domain Structures and Inter-Domain Interactions Defining the Holoenzyme Architecture of Archaeal D-Family DNA Polymerase

**DOI:** 10.3390/life3030375

**Published:** 2013-07-05

**Authors:** Ikuo Matsui, Eriko Matsui, Kazuhiko Yamasaki, Hideshi Yokoyama

**Affiliations:** 1 Biomedical Research Institute, National Institute of Advanced Industrial Science and Technology (AIST), 1-1-1 Higashi, Tsukuba 305-8566, Japan; E-Mails: eriko-matsui@aist.go.jp (E.M.); k-yamasaki@aist.go.jp (K.Y.); 2 School of Pharmaceutical Sciences, University of Shizuoka, Shizuoka 422-8526, Japan; E-Mail: h-yokoya@u-shizuoka-ken.ac.jp

**Keywords:** D-family DNA polymerase, DNA replication, binding domain, molecular structure, hyperthermophilic archaea, *Pyrococcus*

## Abstract

Archaea-specific D-family DNA polymerase (PolD) forms a dimeric heterodimer consisting of two large polymerase subunits and two small exonuclease subunits. According to the protein-protein interactions identified among the domains of large and small subunits of PolD, a symmetrical model for the domain topology of the PolD holoenzyme is proposed. The experimental evidence supports various aspects of the model. The conserved amphipathic nature of the *N*-terminal putative α-helix of the large subunit plays a key role in the homodimeric assembly and the self-cyclization of the large subunit and is deeply involved in the archaeal PolD stability and activity. We also discuss the evolutional transformation from archaeal D-family to eukaryotic B-family polymerase on the basis of the structural information.

## 1. Introduction

Replicative DNA polymerases (DNA Pols) are divided into archaeal-eukaryotic and bacterial types that appear not to be homologous to each other [1,2]. All archaea and eukaryotes, as well as viruses, encode B-family polymerases that are responsible for genome replication [3,4], while bacterial replication is performed by C-family polymerases that are not found in archaea or eukaryotes. All eukaryotes possess four paralogous B-family polymerases, Pols α, δ, ε, and x, involved in DNA replication and repair [5,6]. In addition, Euryarchaeota, a subdomain of archaea, have a distinct type of polymerase, D-family DNA polymerases (PolDs) unrelated to B- or C-family polymerases [7,8,9]. Euryarchaeal DNA-replication is not understood in as much detail as bacteria or eukaryotic replication, whereas all methanogens, key players in greenhouse-gas and bio-fuel production, probably including formation of deep-sea methane hydrate, belong to Euryarchaeota [10].

PolDs, originally discovered from Euryarchaeota [7], were also identified in the archaeal phyla diverged early from the major archaeal phyla Crenearchaeota and Euryarchaeota [11,12,13]. From recent analysis of the evolution of DNA replication apparatus, it is likely that the last common ancestor of archaea had two DNA polymerases of the B-family and one of the D-family [14]. Recent data demonstrated that PolD is an essential DNA polymerase [15]. Indeed, PolD from *Pyrococcus abyssi* (PabPolD) plays an important role in chromosomal replication, together with the B-family DNA polymerase, and is capable of RNA primer elongation [9,16]. We also confirmed that PolD from *P. horikoshii* (PhoPolD) uses RNA primer for DNA synthesis even to a lesser extent than that of the DNA primer, whereas PolB uses only the DNA primer. This strongly suggested that PolD is a key enzyme responsible for lagging strand synthesis. By analogy with the eukaryotic mechanism, it was proposed that PolD completes Okazaki fragments and lagging strand synthesis [9]. 

In contrast to the biochemical properties of PolD, knowledge about the molecular structure of PolD is still scarce. Therefore, in the present article, we review the molecular structure and domain topology of both of the subunits. Especially, we point out that the amphipathic nature of the *N*-terminal ~50 residues of the large subunit is conserved completely in Euryarchaeota and Korarchaeota, and that it possesses a possible function to modify the efficiency of the large-subunit folding and the assembly of the PolD holoenzyme.

## 2. Background

### 2.1. Functional Information for PolD

PhoPolD was proposed to be a dimeric heterodimer (molecular weight: 420 kDa) consisting of two small subunits (DP1s) (PH0123, NCBI accession number NP_142131; 622 amino acids), and two large subunits (DP2s) (PH0121, NCBI accession number NP_142130; 1434 amino acids) [8]. DP2 is the catalytic subunit of DNA polymerase [8], while DP1 is the catalytic subunit of 3'–5' exonuclease. The *C*-terminal domain of DP1 contains five Mre11-like nuclease motifs [17]. Mre11 is a nuclease involved in double-stranded DNA break repair and belongs to the calcineurin-like phosphoesterase superfamily [18]. The second subunits of eukaryotic B-family DNA polymerases (Pols α, β, ε, and x) also show similarities to the Mre11-like exonuclease region, although the catalytic residues of the second subunits are replaced by non-catalytic residues [18,19,20]. The second subunits of the eukaryotic B-family DNA polymerases play a specific role in regulating the first catalytic subunits. Interestingly, it was reported that PolD demonstrated strong DNA polymerase and 3'–5' exonuclease activities when the two subunits were mixed or co-expressed, although each individual subunit demonstrated only a weak activity [17,21]. The domain containing the *N*-terminal 300 residues of DP2 [abbreviated as DP2(1-300); similar descriptions for other fragments will be used in the present manuscript] was reported to be essential for the folding of PolD and is probably the oligomerization domain [22].

The domain topology essential for complex formation of PolD was characterized with the yeast two-hybrid (Y2H) and surface plasmon resonance (SPR) assays [23]. DP2(1-100) interacts with another region in the same subunit, DP2(792-1163), containing the catalytic residues for DNA polymerization, Asp1122 and Asp1124, to form a ring-shaped structure. A putative third acidic-residue involved in the catalytic reaction remains unidentified. Catalytic DP2(792-1163) also interacts with the inter-subunit domain, DP1(1-200). It is noticeable that the polypeptide DP2(792-1163) was expressed as an insoluble form in *E. coli* probably due to its hydrophobicity [23]. As the molecular mechanisms of the protein folding for DP2 were unknown, refolding of the hydrophobic domain DP2(792-1163) harbouring catalytic Asp1122 and Asp1124 residues was investigated by mixing with an equimolar amount of the *N*-terminal domain of DP2 in 3 M urea, and successive stepwise dialysis to remove urea [24]. Before and after dialysis, sampling was carried out and refolding efficiency was examined by SDS-PAGE after removing the precipitate. These results suggest that DP2(1-50) is a minimum and essential element for the refolding of DP2(792-1163) to maintain the complex in soluble form. Furthermore, the complexes DP2(1-100)DP2(792-1163) and DP2(1-300)DP2(792-1163) were recovered completely without any precipitate and were purified completely by gel filtration, indicating that the *N*-terminal 100 amino-acid region, DP2(1-100), is sufficient to refold the catalytic DP2(792-1163) domain as a soluble complex form. According to each band intensity and molecular weight, the molar ratio of DP2(1-100) to DP2(792-1163) and DP2(1-300) to DP2(792-1163) was estimated to be 1 to 1. The molecular weight and the structural uniformity of the purified complex, DP2(1-100)DP2(792-1163) were confirmed by gel filtration [24]. The protein was eluted as a sharp peak and its molecular mass was estimated to be 72 kDa, indicating a uniform dimer, [DP2(1-100)DP2(792-1163)]_2_. Then, the thermostability of the purified dimer, [DP2(1-100)DP2(792-1163)]_2_, was analyzed using a circular dichroism (CD) spectrometer and a fluorometric method between 20 °C and 85 °C [24]. These results indicate that the dimer [DP2(1-100)DP2(792-1163)]_2_ is stable at 85 °C with no dissociation to monomers or drastic conformational changes. The ~50 *N*-terminal residues play essential roles in the dimeric assembly and the self-cyclization of the DP2 subunit.

Using surface plasmon resonance (SPR) assay, we measured the dissociation constant (*K*_D_) of the DP2(1-300) domain against DNA [24]. The *K*_D_ value of the DP2(1-300) domain against 3'-recess DNA is moderate (*K*_D_ = 1.3 × 10^−6^ (M)). Since the *K*_D_ value of the whole PolD molecule to 3'-recess DNA was determined to be 2.7 × 10^−10^ (M), the DP2(1-300) domain seems to play a supplementary role in the DNA-binding mechanism of the dimeric heterodimer PolD. The details of how the domain interacts with DNA need further investigation.

The *C*-terminal domain of DP2, DP2(1164-1434), contains cysteine-cluster (^1289^**C**VK**C**NTKFRRPPLDGK**C**PI**C**^1308^; cysteine residues that are candidates for zinc-binding residues are shown in bold), associates with an inter-subunit domain, DP1(1-200) [23].

### 2.2. Structural Information for Isolated Domains and Fragments

DP2(1-300) has been reported to be essential for the folding of PolD and is probably the oligomerization domain, because the deletion of this part from the PolD holoenzyme caused complete loss of the specific bands on SDS-PAGE analysis of the recombinant-cell extract after heating at 85 °C for 30 min as reported previously [22]. Since the molecular mechanisms of the protein folding and biochemical function of the DP2(1-300) domain were unknown, the crystal structure of DP2(1-300) was determined at 2.2 Å resolution according to the multiwavelength anomalous dispersion (MAD) method [24]. The refined model contains the 48–291 region, although ~50 *N*-terminal residues are disordered. Hereafter, the crystal structure is designated as DP2(48-291). DP2(48-291) has an ellipsoidal shape, and its dimensions are approximately 45 × 30 × 30 Å^3^. DP2(48-291) mainly constitutes an α-helical structure containing nine α helices and three β strands. Three β strands (β1, β2, and β3) form a twisted β-sheet at the center of one face, and the β sheets are surrounded by α4, α5, and α8 helices. The β3 strand and α8 are connected by a 22-residue-long kinked loop, which is located on the surface and is in the vicinity of the β-sheet and α6 helices. The relatively long α8 helix is kinked at Ala-260.

The coordinates of DP2(48-291) were submitted to the web server SSM (Secondary Structure Matching program) to search for other proteins with a similar folding pattern in the PDB. Due to its low structural similarity with other proteins, except for archaeal DP2 (highest Z score = 1.4), the folding of DP2(48-291) was considered to be novel.

**Figure 1:**
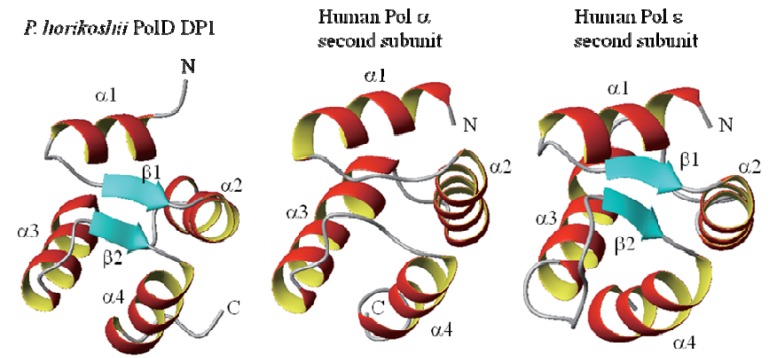
NMR structures of DP1(1-72) of *P. horikoshii* PolD (left) and the *N*-terminal domains of the second subunits of human Pol α and ε (middle, right).

Furthermore, the NMR analysis revealed that region DP1(1-72) contains a folded structure, although the succeeding DP1(73-200) is unfolded [25]. DP1(1-72) part of the domain has only 72 aa. The structure of DP1(1-72) was determined by multi-dimensional NMR methods (Figure 1). The revealed globular structure contains four α-helices and a very short two-stranded parallel β-sheet which was identified in a region connecting the α-helices. Searching the Protein Data Bank (PDB) by the DALI program [26] identified structures similar to DP1(1-72). The similar structures identified with high reliability scores include the *N*-terminal domains of the second subunits of the eukaryotic B-family DNA polymerase, *i.e*., human Pols α and ε (Z-scores 7.2 and 7.1, respectively) [27] (Figure 1). The similar structures also include the δ subunit of the clamp loader γ complex of *E. coli* DNA polymerase III [28], and the domain II of *Thermotoga maritima* RuvB protein [29]. These are classified into AAA+ ATPases, which are chaperonine-like ATPases associated with a variety of cellular activities including DNA replication, recombination, proteolysis, and membrane fusion [30], in which the *C*-domain is similar to DP1(1-72).

The oligomeric state of DP1(1-72) in solution was elucidated by an analytical ultracentrifugation method [25]. Sedimentation equilibrium data in the concentration of 11–350 μM as a monomeric protein showed a slightly curved distribution in a radius^2^ *vs*. ln(A_280_) plot, yielding an average molecular weight of 12.6 kDa indicating an equilibrium between the monomeric and dimeric states (8.5 and 17.1 kDa, respectively). Dimerization is likely to be achieved by hydrophobic interactions as well as electrostatic attractions, although the dimerization mode is not necessarily fixed but is probably rather dynamic. This expands knowledge regarding the domain topology of the holoenzyme. Although this region is connected to the *C*-terminal unstructured portion, a long ~130 amino-acid region, its position in the holoenzyme is probably fixed after association with the remaining part of DP1 and/or DP2. Since DP1(1–200) interacts with PCNA [23], it is possible that the dimer-monomer equilibrium may be influenced by such accessory components, and that the domain presumably possesses a function like a sensor.

The X-ray structure of the complex of the regulatory second subunit (p50) of human DNA Pol δ with the *N*-terminal domain (p66_N_) of the regulatory third subunit was reported (PDB ID 3E0J) [30]. It was also suggested that the second subunit (p50) of human Pol δ lacks the region equivalent to the *N*-terminal domains of the second subunits of human Pol α and ε, and DP1(1-72), and that, instead, the third subunit (p66) supplies an equivalent domain, with a weak sequence similarity [31]. From the structure similarity in the *N*-terminal region and the prominent sequence similarity in the *C*-terminal exonuclease-like region [18,32,33], it is now evident that the DP1 subunit of archaeal PolDs and the second subunit of eukaryotic Pol α and ε are evolutionary related, although that of Pol δ is rather distant. It was recently reported that the first subunit of the eukaryotic Pol ε was derived from chimeric origins between archaeal B-family and D-family polymerases, where only the *C*-terminal zinc finger-like region of the DP2 subunit of PolD was incorporated into the polymerase subunit of Pol ε [14]. We reported that *P. horikoshii* DP1(1–200) interacts with a synthetic peptide corresponding to this zinc finger-like region, DP2(1290–1310) [23]. We suggested, therefore, that, during the evolutionary transformation from archaeal D-family to eukaryotic B-family polymerases the interacting pair of DP1 and the *C*-terminal region of DP2 is conserved, probably in order to maintain the holoenzyme structure [25].

## 3. A Model for the PolD Holoenzyme

**Figure 2:**
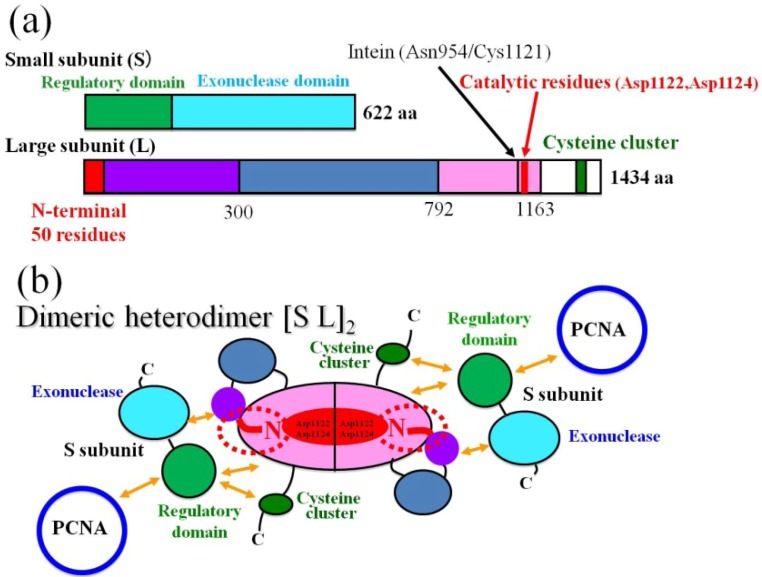
Domain structure and topology of the PhoPolD holoenzyme. (**a**) Schematic maps of the domains for the small and large subunits. The mini-intein insertion site and the catalytic center for polymerization in the large subunit (L) are shown with arrows and vertical lines, respectively; (**b**) The domain topology of dimeric heterodimer. Each domain is colored in the same manner as (a). The ~50 *N*-terminal residues of the large subunit are depicted with dotted red circles. The red letters “N” in the dotted circles indicate the *N*-terminus of the large subunit. The interactions between each domain are shown with arrows in orange [23]. The replication factor, proliferating cell nuclear antigen is abbreviated as PCNA.

We reported the domain topology essential for complex formation and interaction with other proteins, which was characterized with the yeast two-hybrid (Y2H) and surface plasmon resonance (SPR) assays [23]. Refolding of the catalytic domain DP2(792-1163) was investigated by mixing with an equimolar amount of the *N*-terminal domain of DP2. The dimer, [DP2(1-100)DP2(792-1163)]_2_ was reported to be heat stable and a uniform molecule [24]. The results of the refolding experiments suggest that the dimer, [DP2(1-100)DP2(792-1163)]_2_ forms a central core of the dimeric large subunit, (DP2)_2_, in which DP2(1-50) is an essential element for the refolding of DP2(792-1163) to maintain the complex in soluble form. On the basis of the results, here we propose a revised domain topology of the PolD holoenzyme, probably associated in a symmetric manner as shown in Figure 2. In the symmetric model, two small subunits (DP1) are associated with a dimeric large subunit, (DP2)_2_ by multiple protein-protein interactions shown with arrows in orange (Figure 2b). It is noticeable that the two small subunits (DP1) are also placed in the anti-parallel direction from the central two-fold axis in Figure 2b.

## 4. The Amphipathic Nature of the ~50 *N*-Terminal Residues Conserved Completely in PolD of Archaea

**Figure 3:**
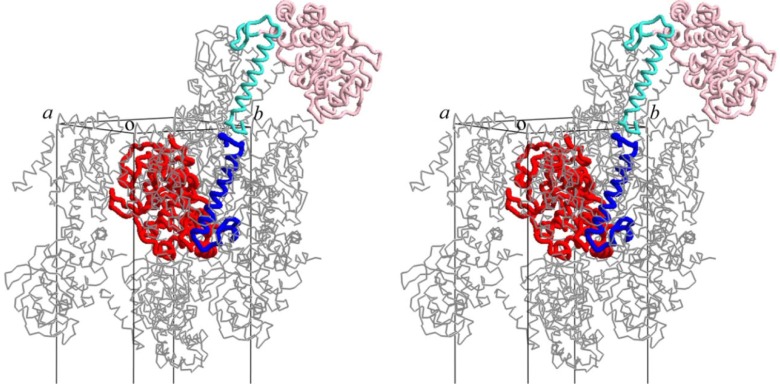
Crystal packing of DP2(48-291) with modeled α-helices DP2(1-47) is shown in stereo view. One DP2(48-291) molecule and the tentative model of DP2(1-47) are shown in red and blue, and symmetry-related molecules are shown in pink and cyan, respectively.

According to secondary-structure prediction using the amino acid sequence and the PSIPRED program [34], the region between residues 8 and 33 of DP2 is likely to form an α-helix. In order to estimate how the disordered ~50 *N*-terminal residues of DP2 form the dimeric assembly, we manually built a model of residues 1 to 47 in the crystal packing of DP2(48-291). The modeled α-helices were fitted well in the remaining space of the crystal packing for DP2(48-291) as shown in a stereo view of Figure 3, suggesting one possible conformation of the ~50 *N*-terminal residues after trapping in the crystal lattice. One DP2(48-291) molecule and the tentative model of DP2(1-47) are shown in red and blue, and symmetry-related molecules are shown in pink and cyan, respectively.

**Figure 4:**
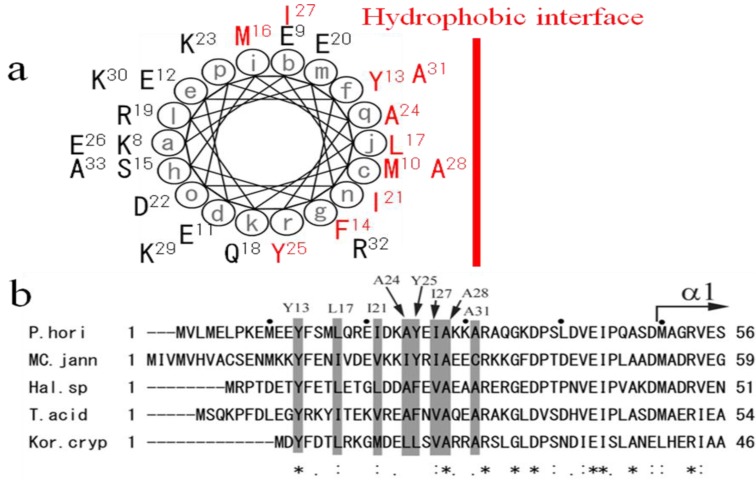
The amphipathic nature of the *N*-terminal extremity conserved well in the large subunits DP2 of archaea. (**a**) Helical wheel projection of DP2(8-33). Hydrophobic amino-acid residues are colored in red. A hydrophobic interface is indicated with a red vertical line; (**b**) Sequence alignment and the hydrophobic profile conserved in the ~50 *N*-terminal residues of archaeal DP2. The hydrophobic profile is emphasized with gray shading in the alignment. The eight hydrophobic residues in the conserved region between residues 8 and 33 of PhoDP2 are depicted on the upper side of the alignment. The starting point of the secondary element (α1 helix) of PhoDP2 is also indicated with an arrow. The figure was produced with EMBL-EBI tool ClustalW. The asterisks indicate identical residues, and the period and colon indicate similar residues among species. The sequences are from five archaeal species, *P. horikoshii* (*P. hori*, NCBI accession number: NP_142130), *Methanococcus jannaschii* (MC. jann, accession number: U67603-3), *Halobacterium* sp. NRC-1 (*Hal*. sp., accession number: AE005116-6), *Thermoplasma acidophilum* (T. acid, accession number: AL445063-36), and *Korarchaeum cryptofilum* (Kor. cryp, NCBI accession number: NC_010482).

Since the disordered ~50 *N*-terminal residues was likely to form an α-helix with complex formation according to secondary-structure prediction and molecular modeling, helical wheel analysis of DP2(8-33) was carried out with the DNASIS-Mac ver 2.0 software. As shown in Figure 4A, half of the wheel is hydrophobic and covered with 11 hydrophobic residues (Met10, Tyr13, Phe14, Met16, Leu17, Ile21, Ala24, Tyr25, Ile27, Ala28, Ala31), and the other side is hydrophilic [24]. Since these hydrophobic residues are conserved well in all euryarchaeal DP2s as shown in Figure 4B, the α-helix DP2(8-33) should associate with hydrophobic domain DP2(792-1163) on the hydrophobic interface. We have already found three hydrophobic regions in the *C*-terminus of DP2(792-1163) [23]. One or more hydrophobic regions forming the catalytic center might associate with the amphipathic α-helix (8-33). Interestingly, it was recently reported that the mutant of (G)-PYF box motif located at one of the three hydrophobic regions forming the catalytic center of PabPolD from *P. abyssi* were rapidly degraded, suggesting the (G)-PYF box inside the hydrophobic domain DP2(792-1163) has a major role in PolD stability and in polymerase activity [35]. We have previously reported that the deletion of DP2(1-300) from the PolD holoenzyme made it unable to detect the PolD proteins from the recombinant-cell extract after heating, indicating the major role of DP2(1-300) in PolD stability [22]. We also reported that its ~50 *N*-terminal residues play a key role in PolD folding and stability. The (G)-PYF box motif might be a counterpart to assemble with the putative α-helix, DP2(8-33), although further work would be necessary to confirm the interaction. Since PolDs are essential in DNA replication and repair [15] and the amphipathic nature of the ~50 *N*-terminal residues is conserved completely in Euryarchaeota and Korarchaeota as shown in Figure 4B, mutations to modify the amphipathic nature might change the phenotypes of these archaea, especially in PolD stability and in polymerase activity. The *N*-terminus of DP2 seems to be a major target for the DNA replication control.

## 5. Conclusions

Archaea-specific D-family DNA polymerase (PolD) forms a tetramer consisting of two large polymerase subunits (DP2) and two small exonuclease subunits (DP1). PolDs, originally discovered from Euryarchaeota, were also identified in the putative phyla Nanoarchaeota, Thaumarchaeota (formally mesophillic Crenarchaeota), and Korarchaeota, which may have diverged early from the major archaeal phyla Crenearchaeota and Euryarchaeota.

Interestingly, the *C*-terminal part of DP1, eukaryotic Mre11-like nuclease domain, shows low but significant homology to the non-catalytic second subunit of eukaryotic B-family DNA polymerases (Pols α, δ, and ε). We reported that the *N*-terminal domain of *Pyrococcus horikoshii* DP1 interacts with a synthetic peptide corresponding to the *C*-terminal zinc finger-like region of the DP2 [23]. The NMR structure analysis of the *N*-terminal domain of DP1 suggested that the interacting pair of DP1 and the *C*-terminal zinc finger-like region of the DP2 are conserved during the evolutionary transformation from archaeal D-family to eukaryotic B-family polymerases, probably in order to maintain the holoenzyme structure [25].

We reported the *N*-terminal (1–300) domain structure determined by X-ray crystallography, although ~50 *N*-terminal residues were disordered [24]. The determined structure consists of nine α helices and three β strands. We also identified the DNA-binding ability of the domain by SPR measurement, suggesting that the structure shows a novel DNA-associating fold. Refolding of the catalytic domain for the large subunit (DP2) by mixing with its *N*-terminal domain of DP2 suggested that the disordered *N*-terminus (~50 residues) play a key role in self-cyclization and homodimeric assembly of DP2. According to the molecular structure of the *N*-terminal region of DP2 and the symmetrical topology model of the PolD holoenzyme, the amphipathic nature of the *N*-terminus interacting with the active center of DP2 might be a potent tool to control the archaeal PolD stability and polymerase activity.
